# Preparation and Evaluation of Microencapsulated Fast Melt Tablets of Ambroxol Hydrochloride

**DOI:** 10.4103/0250-474X.56028

**Published:** 2009

**Authors:** S. Jacob, A. Shirwaikar

**Affiliations:** Department of Pharmaceutical Sciences, S. Bhagwan Singh (PG) Institute of Biomedical Sciences and Research, Balawala, Dehradun-248 161, India; 1 Department of Pharmaceutics, Gulf College of Pharmacy, Ajman, Dubai, UAE

**Keywords:** Aqueous colloidal polymer dispersion, microencapsulation, microsphere pectin, taste masking

## Abstract

Natural resources in general and plant materials in particular are receiving more attention due to their safety as pharmaceutical excipients. Present work assessed the potential of a natural polysaccharide, pectin to mask the bitter taste of ambroxol hydrochloride, by microencapsulation technique, and its possibility to formulate as a fast disintegrating dosage form. Taste masking is an important developmental challenge in fast dissolving drug delivery system since it dissolves or disintegrates in the patient's mouth in close proximity to the taste buds. The prepared microspheres by emulsion solvent evaporation technique possessed good sphericity, smooth surface morphology, uniform and narrow size distribution (10-90 μm), when analyzed by scanning electron microscopy, laser diffraction and optical microscopy. Method of preparation has influenced the particle size and drug loading efficiency. Drug-polymer compatibility was confirmed by Fourier transform infrared spectroscopy and thin layer chromatography. DSC and X-ray diffraction studies revealed that the drug was dispersed inside the microspheres in the form of an insoluble matrix. The formation of microspheres was affected by glass transition temperature of the polymer, surfactant, type of plasticizers, volume of internal phase, stirrer speed etc. Fast dissolving tablets were prepared by the modification of melt granulation technique. The resulting granules were found to melt fast at body temperature, have smooth mouth feel and good physical stability. This study demonstrated that pectin could be a right choice in developing patient favored formulations for bitter drugs and can be utilized in fast disintegrating dosage forms as well.

Due to increased life expectancy, the elderly constitute a major portion of the world population today. These people will experience deterioration of their physiological and physical abilities like dysphagia. It is also common in young children because of their underdeveloped muscular and nervous systems. Fast melt dosage forms i.e., one that disintegrates and/or dissolves rapidly in the saliva without the need for water, provides an exciting opportunity to fulfill these medical needs. Sometimes they are designed to be absorbed through the buccal and oesophageal mucosa as the saliva passes into the stomach. Currently, there are seven fast dissolving/disintegrating technologies in the world market. They are Zydis (R. P. Scherer, Inc), Wowtab (Yamonouchi Pharma Technologies, Inc), Orasolv and Durasolv (Cima Labs, Inc), Flash dose (Fuisz technologies Ltd.), Flashtab (Prographarm Group) and Oraquick (K. V. Pharmaceutical Co., Inc).

Melt granulation is a single step technique converting fine powders into granules of various sizes and more or less regular spherical shape. US Patent No, 855,326, describes a melt spinning process with “cotton candy” fabricating equipment using a spinnable carrier agent such as sugar combined with a medicament. One disadvantage of the above process is that melt spinning step is required in addition to direct compression technique. Preparation of spherical beads without the use of any solvents by a novel tumbling melt granulation technique has been described by Maejima *et al*[1]. Preparation of melt granulation in a laboratory scale high shear mixer and process variables for screening of high shear mixer melt granulation by using an asymmetrical factorial design have been studied[2,3]. Karali *et al.* have described a wet granulation method for the preparation of fast melt tablets using gums or cellulosic materials and blending with a saccharide of low moldability to result in the formation of granules[4]. Fast melt multiparticulate formulation for oral delivery has been patented by Tobyn *et al*[5]. Fast dissolving dosage forms having reduced friability have been patented by Pruss *et al*. In the above method low melting components like polyethylene glycol and triglycerides were used[6].

Fast dissolve dosage forms dissolve or disintegrate in the patient's mouth in close proximity to the taste buds. Unless the drug is tasteless or does not have an undesirable taste, the use of taste masking techniques becomes critical to patient acceptance. So this is one of the important development challenges in the fast dissolving drug delivery system. The taste of the active ingredient could be masked by microencapsulation technique. Microencapsulation techniques based on water insoluble polymer carriers require organic solvents to solubilize the polymers[7–9]. However, the safety hazards, toxicity and high costs associated with organic solvents make the use of an organic solvent free system desirable. Hence we decided to entrap a water soluble highly bitter model drug using pectin, in relatively organic solvent-free environment. The natural polymer pectin is economical and widely available. Pectins are non starch, linear polysaccharides extracted from the cell walls of plants. They are predominantly linear polymers of α (1-4)-linked D-galactouronic acid residues interrupted by 1,2 linked L-*γ* Rhamnose residues. Pectin has a few hundreds to about one thousand building blocks per molecule, corresponding to an average molecular weight of about 50 000 to 180 000. It is non-toxic, approved by FDA (inactive ingredients database) and almost totally degraded by colonic bacteria but not digested by gastric or intestinal enzymes. Microspheres of water soluble carriers such as albumin have been prepared by the emulsification of an aqueous drug carrier solution into an external oil phase to form a w/o emulsion with the formation of microparticles after water removal[10]. Organic solvent free polymeric microspheres have been prepared from an aqueous colloidal polymer dispersion by using a w/o emulsion technique[11]. Water soluble drugs can be encapsulated by either an organic phase separation or a non-aqueous solvent evaporation technique; however, the drugs have to be insoluble in the solvent used[12]. Majority of the taste masking polymers are enteric in nature and require organic solvent based microencapsulation technique. This might compromise on bioavailability and can increase the cost of this delivery system. Water based pectin microspheres could mask the taste of the drug sufficiently long enough in the oral cavity of fast dissolving tablets followed by fast and complete release as for any immediate release dosage form.

## MATERIALS AND METHODS

Polyethylene glycol 1000 (Koch-Light laboratories Ltd, Coinbrook Bucks, UK), xylitol, liquid paraffin (Merck India Ltd, Mumbai, India), dextrose, sodium lauryl sulphate and low methoxy pectin, croscarmellose (S. D. Fine Chem, Mumbai, India), colloidal silicon dioxide, *n*-hexane (Nice Chemicals Pvt. Ltd, Kochi, India), microcrystalline cellulose (Avicel® PH101, FMC Corporation, Philadelphia, USA), ambroxol hydrochloride Tablets India Ltd, Chennai, India; Aristo Pharmaceuticals, Madhy Pradesh, India.

### Preparation of microspheres:

Pectin, finely ground in a mortar and passed through sieve No. 120 was used for the preparation of polymer dispersion in 2, 4, 8 or 15 ml of distilled water. For e.g., in the first ratio, pectin was used at a concentration of 10% w/v and to this, 1 ml of 0.1% w/v of sodium lauryl sulphate solution was added to prevent the aggregation of microspheres. Various concentrations of ambroxol hydrochloride were dispersed into this to obtain different ratios viz 1:1, 1:2, 1:4, 1:7, and 1:10. The resulting polymeric dispersion was emulsified into an external light liquid paraffin oil phase. The mixtures were stirred at constant stirring speed (3000 rpm) and agitated in a water bath at various temperatures like 60, 70, or 80° at controlled humidity (30% RH) by means of dehumidifier to evaporate the water. After cooling the micro particle/oil suspension to room temperature, the microspheres were collected by decantation, washed with *n*-hexane to remove excess of oil from microsphere surface and dried in an oven at 40° for 5 h.

### Size and size distribution:

The microspheres were mounted in liquid paraffin were examined using an optical microscope (Olympus, Japan, eye piece/objective 10X/100X) and laser diffraction technique (Malvern Instruments Ltd. Malvern, UK). The size and size distribution of the microspheres were determined from a total of 100 microspheres of 10 cycles. The dispersant used was cyclohexane. Particle size and shape parameters like surface weighted mean D [3,2], volume weighted mean D [4,3], specific surface area were determined. Particle size distribution parameters like d (0.1), d (0.5), and d (0.9) were analyzed.

### Scanning electron microscope (SEM) studies:

**Fig. 1:**
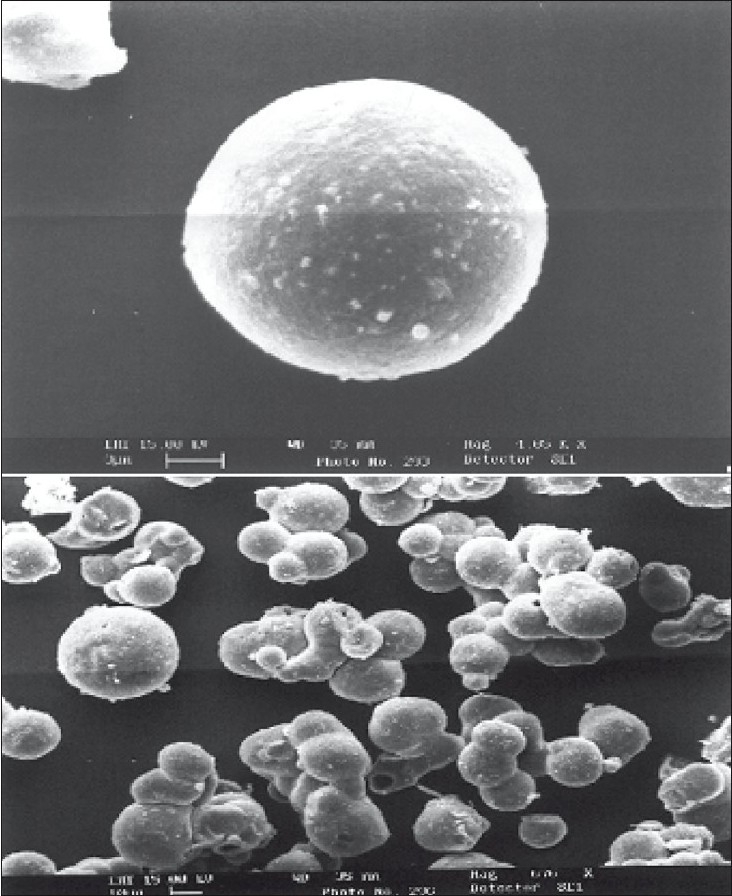
Scanning electron micrograph of microspheres. Scanning electron micrograph of a single and group of pectin microspheres, drug:pectin ratio is 1:7.

SEM photographs were taken for the pectin microspheres prepared by emulsion solvent evaporation technique and are depicted in fig. 1. The sample was mounted on an aluminum stud using double adhesive carbon tape. Microspheres were coated using Poloron E5100 SEM, coating system. Scanning was done using LEO Electron microscopy Ltd, Cambridge; UK. The micrographs were recorded at HT 15 KV accelerating voltage using LEO 435VP.

### Differential scanning calorimetry (DSC) studies:

**Fig. 2:**
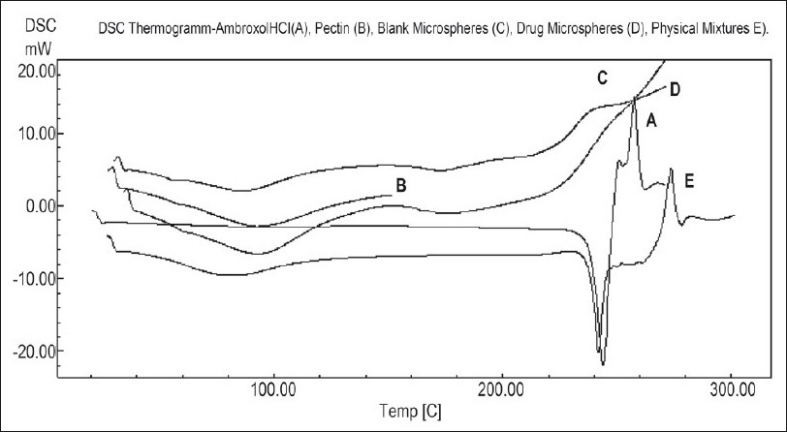
DSC Thermograms of drug microspheres and physical mixtures. DSC Thermograms of A. ambroxol, B. pectin, C. drug microspheres and D. physical mixtures

Differential scanning calorimetry (DSC) was performed on pectin, pure drug, placebo microparticles and the drug loaded microspheres as given in fig. 2. DSC measurements were done on a Shimadzu DSC-60 and samples were heated at the rate of 10° min^−1^. The samples were heated in an aluminum cup up to 250°. The reference used was indium.

### Evaluation of taste masking ability:

The taste masking ability was determined by healthy volunteers in a double blind study (n=3). Taste evaluation began immediately after administration and continued for 60 sec. The taste masking period was calculated /expressed as the difference between the administration time and onset time of bitter taste[13].

### Determination of drug loading:

An accurately weighed amount of microspheres dispersed in an appropriate volume of distilled water was agitated in an orbital shaker until the microspheres were completely dissolved[14]. The aliquot sample was passed through a Whatmann filter paper and diluted with distilled water. The drug content was determined spectrophotometrically at a wavelength 245 nm. The drug content was expressed as the amount of drug encapsulated in a unit weight of microspheres. The drug content of each sample was determined in triplicate and results averaged.

### Drug release study:

The dissolution profiles of microspheres were obtained by placing 20 mg of microparticles in a dialysis bag and then introducing it into a glass beaker containing 50 ml of 0.1 M phosphate buffer (pH 7.4) maintained at 37°. The receptor medium was stirred with magnetic bead at 50 rpm. The samples were withdrawn at predetermined intervals and replaced with the same amount of buffer to maintain sink condition. The amount of drug released was determined by UV spectroscopy.

### Interaction between pectin and drug:

The drug-excipient compatibility was studied using Fourier transform infrared spectroscopy and thin layer chromatography using drug-excipient ratio 1:10 as encapsulation efficiency was found to be minimum at this ratio as shown in Table 1.

**TABLE 1:** EFFECT OF POLYMER RATIO ON THE AMBROXOL HCL LOADING OF PECTIN MICROSPHERES*

Drug:Polymer	Theoretical drug loading (%)	Actual loading (%)	Encapsulation efficiency (%)
1:1	50.0	38.38±5.78	76.76±4.67
1:2	33.33	26.59±7.89	79.77±3.67
1:4	20.0	17.87±4.83	89.35±3.78
1:7	12.5	11.90±3.26	95.20±3.98
1:10	9.09	6.09±7.90	66.99±5.41

* Each value is the means of n=3 determinations

### Preparation of fast melt granules:

Initially a mixture of xylitol and dextrose was prepared and passed through sieve 80. It was then blended with colloidal silicon dioxide and microcrystalline cellulose. Specified amount of polyethylene glycol 1000 was melted at 50°. It was then added gradually to the above mixture and mixing was continued at high speed until granules were formed. They were then allowed to congeal. The congealed granules were passed through sieve 60 and vacuum dried for 2 h to remove moisture. These fast disintegrating granules were used for preparing tablets.

### Flowability of powders:

The static angle of repose was measured according to the fixed funnel and free standing cone method[15]. The bulk density of the mixed powders before compression was calculated by determining the Hausner's ratio and Carr's index from the poured and tapped bulk densities of a known weight of sample using a measuring cylinder and the following formula[16,17]. Hausner's ratio = D_p_/D_t_, Carr's index = (D_p_−D_t_)/D_p_×100, where D_p_ (poured density) = weight/Vp (poured volume), D_t_ (tapped density) =weight/V_t_ (tapped volume).

### Preparation of tablets:

The composition of tablet is presented in Table 2. The composition was compressed into flat tablets with 10 mm diameter using a single punch tablet machine at a fixed compression force. The punches and die were lubricated with a small amount of magnesium stearate using a cotton swab preceding compression. The tablets were stored at 25° and 34% relative humidity for one week in a desiccator. The relative humidity of the desiccator was controlled by the use of a saturated solution of magnesium chloride hexahydrate.

**TABLE 2:** FORMULATION OF A FAST MELTS TABLET PREPARED BY DIRECT COMPRESSION

Ingredients	mg/tablet
Ambroxol hydrochloride microparticles (equivalent to 30 mg ambroxol HCl)	240.56
Croscarmellose sodium	25.0
Corn starch	30.0
Aspartame sodium	9.5
Americant mint	3.5
Silicon dioxide	3.0
Magnesium stearate	2.1
Fast dissolving granulation	186.34
Total	500.0

### Measurement of tablet tensile strength and friability:

The tablet crushing load, which is the force required to break a tablet into halves by compression in the diametric direction, was measured using a Pfizer tablet hardness tester. Tablet's friability was measured using Roche friabilator USP at 25 rpm for 4 min.

### Measurement of disintegration time *in vitro* and *in vivo*:

The disintegration test was performed using an IP 85 disintegration apparatus, with distilled water at 37±0.5°. All tablet property values are shown as averages of five determinations. The complete disintegration time in the mouth was measured in five healthy volunteers. The end point for the disintegration in the mouth is the time when the tablet placed on the tongue disintegrates until no lumps remain. While testing, the volunteers kept the tablets motionless on their tongues[18].

### Measurement of tablet porosity:

The porosity of the tablet was calculated from bulk and true tablet volume. It was calculated from the measured tablet diameter, thickness, true density of powder using the following equation E= 100 (1-V_t_/V_b_)[19]. The diameter and thickness of the tablet were measured with a micrometer. The true density of the powder was determined using a helium pycnometer (AccuPyc 1330, Micrometitics Instrument Inc., Norcross, GA).

### Wetting time and water absorption ratio:

A piece of tissue paper folded twice was placed in a small Petri dish (i.d.=6.5 cm) containing 6 ml of water. A tablet was placed on the paper and the time required for complete wetting was then measured. The water absorption ratio, R, was determined using the following equation, R=W_a_−W_b_/W_b_×100, where W_b_ is the weight of the tablet before water absorption and W_a_ is the weight of the tablet after water absorption[20]. The results were the average of five measurements.

### Dissolution test:

Dissolution test was carried out in 900 ml of 0.1N HCl at 37±0.5° in a dissolution tester USPXXIV with a paddle rotation at 50 rpm. An aliquot of dissolution medium was withdrawn at various time intervals and absorbance was measured at 245 nm spectrophotometrically. An equal volume of the dissolution medium was added to the beaker to maintain sink condition. Dissolution was carried out for all designed formulations and conventional marketed tablet[21].

### *In vivo* oral absorption test:

*In vivo* test was performed to study the oral absorption of the prepared ambroxol hydrochloride after each formulation was administered to three healthy volunteers, by keeping the tablet in the oral cavity until disintegration[22]. The subjects then rinsed their mouth with an aliquot of distilled water. It was quantified by using UV spectrophotometer to determine the amount remaining in the oral cavity. The amount of drug absorbed through the oral mucosa was calculated by subtracting the amount from the initial amount.

## RESULTS AND DISCUSSION

**Fig. 3:**
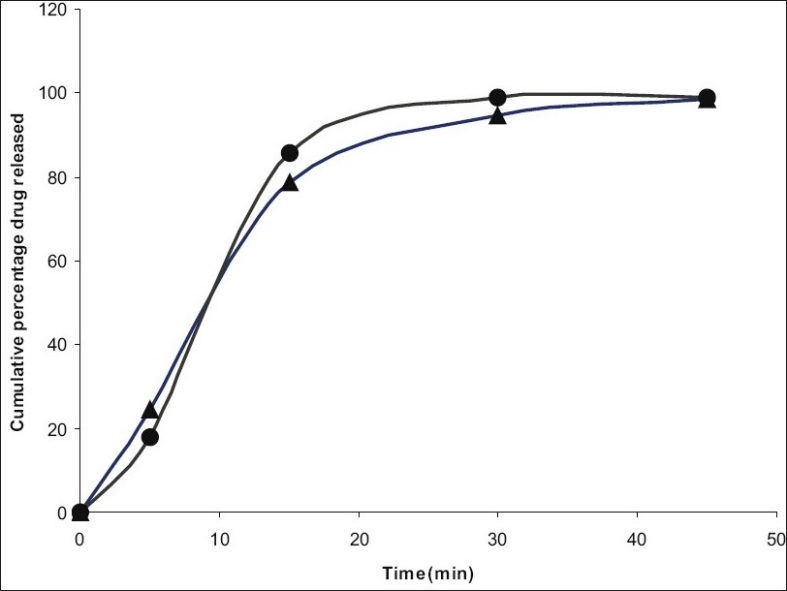
Dissolution profile of ambroxol from conventional tablet and selected microencapsulated fast melt tablet. Dissolution profile of ambroxol from conventional tablets (–▲–) and selected microencapsulated fast melt tablet (–*–) (B_4_) in 0.1N HCl. Each value is a mean±SD of 3 determinations

A prerequisite for the successful preparation of microparticles is the compatibility between the polymer and the drug. Drug polymer interaction when studied by FT-IR and thin layer chromatography showed no drug:excipient interaction. Oral sustained delivery of ambroxol from its *in situ* gelling calcium pectinate formulations have been prepared successfully[23]. In our study polymer dispersions were stabilized against premature flocculation and coalescence by the addition of an anionic surfactant like sodium lauryl sulphate. The functional properties of pectin were determined by the percentage of carboxyl groups that have been esterified or amidated and denoted as the degree of esterification (DE) and degree of amidation (DA), respectively. Milling can reduce the molecular weight of pectin without modifying its degree of esterification (DE), possibly by random scission of the main chain by mechanical attrition[24]. Therefore, the polymer chains formed in solution are thin, more flexible and uncoiled almost instantaneously due to changing shear rates. Hence in our study the microparticle formation may probably be due to the diffusion of water into the oil phase followed by the evaporation at the oil/water interface. Pectin, a polyelectrolyte is negatively charged at neutral pH and approaches zero charge at acidic pH. The pH of the formulation therefore affects the degree of ionization of pectin molecules and its electrostatic interaction with the drug. The pH of the pectin formulation is around two, with a charge that is slightly negative or nearing zero. At this pH, ambroxol will be in ionized state since its pKa value is around 8.2 and this may be responsible for entrapment of ambroxol hydrochloride, a cationic drug by ionic complexation. The gelation of polysaccharides like pectin takes place in the presence of hydrogen ion. Drugs containing pectin beads have been successfully prepared using the ionotropic gelation method, thereby proving the usefulness of pectin as a matrix for the encapsulation of both cationic and anionic drugs[25]. The ion exchanging properties of pectin vary depending on the degree of esterification of the carboxyl groups, the method of preparation and the distribution of free and esterified carboxylic acid on the macromolecule. Therefore, different interactions with drugs appear to be possible particularly when the drugs have a cationic character. Various formulation and process variables were investigated to understand and chracterize the formation of microparticles. The encapsulation efficiencies with various ratios of drug and polymer are found to be in the range of 70-95% as shown in Table 1. The microparticles were isolated as spherical, non-agglomerated, free flowing powders with multiple nuclei in the micrometer size ranges. Scanning electron micrograph of pectin microspheres have shown in fig. 1. Most of the microspheres were found to be spherical in shape and few being slightly elongated. As can be seen from these micrographs, few microspheres exhibited porous surface structure and particles embedded on the surface. This might be responsible for the increase in dissolution as shown in fig. 3. The physical state of the drug inside the pectin microspheres was assessed by differential thermal analysis. DSC thermograms of ambroxol, pectin, physical mixtures, blank and drug-microspheres are shown in fig. 2. Under experimental conditions, no DSC peak was observed for the pectin and drug free microspheres. For the physical mixtures the endothermic peak was similar to that of drug. This might be due to the crystalline form of ambroxol existed in the physical mixtures. For the drug loaded pectin microspheres, however, no peak was observed, even when drug content was high. This result indicated that the drug was dispersed as an insoluble marix. The particle sizes were found to depend on the internal phase volume as shown in Table 3. This might be due to the increased swelling due to the higher water binding capacity of pectin[26]. Particle size and shape parameters like surface weighted mean D[3,2], volume weighted mean D[4,3], specific surface area are determined. Particle size distribution parameters like d(0.1), d(0.5), d(0.9) was also analyzed as given in Table 4.

**TABLE 3:** EFFECT OF THE VOLUME OF THE INTERNAL PHASE ON THE AMBROXOL HCL LOADING AND PARTICLE SIZE OF PECTIN MICROSPHERES (DRUG: PECTIN 1:7)

Internal phase (ml)	Theoretical drug loading (%)	Actual drug loading(%)	Encapsulation efficiency(%)	Mean Particle size (μm±SD)
2	12.5	11.67	93.36±1.13	10.7±0.98
4	12.5	12.01	96.08±0.11	22.37±0.08
8	12.5	10.72	85.76±0.35	80.88±0.97
15	12.5	9.98	79.84±0.49	90.83±0.79

**TABLE 4:** EFFECT OF THE VOLUME OF THE INTERNAL PHASE ON THE PARTICLE SIZE OF PECTIN MICROSPHERES (DRUG: PECTIN 1:7).

Internal phase (ml)	Specific surface area (m^2^/g)	Surface weighted mean D[3,2]	Volume weighted mean D[4,3]	Mean particle size, d(0.5) (μm±SD)
2	0.604	9.93	40.46±0.32	37.26±0.89
4	0.238	25.16	103.31±0.43	70.05±0.45
8	0.218	27.56	150.14±0.67	93.82±0.97
15	0.053	112.19	302.19±0.75	172.5±0.79

The encapsulation efficiency of the microparticles was not affected by the selected temperature of the external phase as shown in Table 5 which was in agreement with the findings of Bodmeir *et al.* but it is necessary that the humidity of the external environment should be controlled for the rapid evaporation of water[9]. The temperature selected was found to depend on glass transition temperature and minimum film formation temperature of the polymer. Faster coacervation, reduced stirring time and maximum product yield occurred at an optimum temperature. The evaluation of the unpleasant taste of ambroxol hydrochloride revealed that the palatability and taste of the drug were significantly improved by microencapsulation. Relative assessment of taste using a neutral pH medium was done to establish an approximate baseline for early time point dissolution value. The delay in drug release only needs to be long enough to pass through the oral cavity followed by fast and complete release as for any immediate release dosage form[27]. The dissolution profiles of an ideal taste masked immediate release product and pectin microspheres are given in fig. 3. The present study shows that water soluble polymers like pectin are ideal for orally disintegrating solid dosage forms.

**TABLE 5:** EFFECT OF THE TEMPERATURE OF OIL PHASE ON AMBROXOL HCL LOADING IN TO PECTIN MICROSPHERES

Temperature (°C)	Theoretical drug loading(%)	Actual drug loading (%)	Encapsulation efficiency (%)
60	12.5	11.98±0.11	95.84±0.45
70	12.5	12.04±0.40	96.32±0.78
80	12.5	11.95±0.61	95.60±0.67

Effect of the temperature of liquid paraffin oil phase on ambroxol HCl loading in to pectin microspheres with a 1:7 ration of drug to pectin

Fast dispersing formulation commonly called fast melting tablets also offer advantages over other dosage forms such as effervescent tablets, chewing gum, extemporary suspensions, which are commonly used to enhance patient compliance[28]. Because of their high aqueous solubility and sweetness, which imparts a pleasing mouth feel and good taste, nearly all formulations for rapidly dissolving tablets contain sugar-based materials like xylitol, mannitol and sorbitol[29]. Of the above mentioned sugar alcohols, xylitol is preferred because it has a good taste and dissolves most rapidly in the oral cavity due to high negative heat of solution. When xylitol is used in combination with mono or disaccharides, tablets with high tensile strength and short oral cavity dissolution can be obtained even when compressive pressures exceed 300 kg[30]. So in this process we have used the combination of xylitol and dextrose, both being highly soluble sugars. The tablets produced with dextrose are less friable and have a tendency to harden on aging[31]. It improved the hardness and did not compromise the disintegration time as shown in Table 6. Bi *et al*. used microcrystalline cellulose as disintegrant to prepare rapidly disintegrating tablets. It has a high internal porosity and a large surface area due to randomly aggregated filamentous microcrystals. It provides highly absorbent, lubricant and moisture retaining and distributing properties that are essential to the extrusion process as pelletization aid[32]. Moisture activated dry granulation process was described by Ullah *et al*[33]. In this procedure microcrystalline cellulose is added to sorb the small amount of moisture present. No traditional drying step is involved. The granulation tends to be nondense, with a relatively small particle size. These attributes of microcrystalline cellulose are used to prepare granules with polyethylene glycol 1000, which has a semisolid consistency. In addition colloidal silicon dioxide has been included, which has a large specific surface area (200-400 m^2^/g) and strong adsorbent capacity[34]. It is also used as a tablet disintegrant and as an adsorbent dispersing agent for liquids in powders or suppositories. Even at a very low compressive pressure, there is always damage to the coating membrane. Nevertheless, by the appropriate selection of the excipients, it is possible to achieve a formulation to ensure a minimum damage to this coating. The good tabletting properties of Avicel for tabletting microcapsules have been pointed out in the literature[35]. A combination of excipients with low yield pressure value like polyethylene glycol and microcrystalline cellulose is proposed as a suitable excipient mixture for coated particles[36]. To circumvent the problems of capping and lamination and structural failures pectin types are blended with plastically deforming highly compactable microcrystalline cellulose[37]. Therefore the mixture of above excipients makes an ideal combination for fast melting tablets. Attributes such as fast tablet dissolution, good mouth feel and good tablet physical stability are of greater importance than high or low value tablet hardness. A low melting point component which melts or softens at body temperature produces smooth feel and masks the grittiness of the insoluble ingredients. Because of the combination of melting, disintegration of the tablet matrix, and dissolution of the water-soluble excipients, dry mouth feel does not occur.

**TABLE 6:** FORMULATION AND HARDNESS, DISINTEGRATION TIME AND DISSOLUTION CHARACTERISTICS OF BATCHES IN A 3^2^ FULL FACTORIAL DESIGN

Batch No.	Variable level in coded form		Hardness of tablets (kg/cm^2^)	Disintegration Time (sec)	t_80_ (min)
	X_1_	X_2_	Y_1_	Y_2_	Y_3_
B_1_	−1	−1	3	65	30
B_2_	−1	0	3	52	28
B_3_	−1	1	3	60	35
B_4_	0	−1	3.5	30	24
B_5_	0	0	3	45	34
B_6_	0	1	3.5	46	40
B_7_	1	−1	2	29	29
B_8_	1	0	2	50	30
B_9_	1	1	2	54	32

All batches contained 500 g of polyethylene glycol 1000. Coded values of −1, 0, and 1 stand for, X_1_ ratio of 0:2000 and X_2_ ratio of 20:2000, X_1_ ratio of 1000:1000 and X_2_ ratio of 40:1500 and X_1_ ratio of 2000:0 and X_2_ ratio of 60:1000, respectively. X_1_ is the ratio of xylitol to dextrose and X_2_ is the ratio of colloidal silicon dioxide to microcrystalline cellulose.

A 3^2^ full factorial design was constructed to study the effect of ratio of xylitol to dextrose (X_1_) and ratio of colloidal silicon dioxide to microcrystalline cellulose (X_2_) on the dependent variables like hardness (Y_1_), disintegration time (Y_2_) and t_80_ (Y_3_). A statistical model incorporating interactive and polynomial term was utilized to evaluate the response= b_0_+b_1_ X_1_+b_2_ X_2_+b_12_ X_1_ X_2_+b_11_ X_1_^2^+ b_22_ X_2_^2^ -(1), where the dependent variable, b_0_ is the arithmetic mean response of nine runs, and b_1_ is the estimated coefficient for the factor X_1_. The main effects (X_1_ and X_2_) represent the average result of changing one factor at a time from its low to high value. The interaction terms (X_1_ X_2_) show how the response changes when two factors are simultaneously changed. The polynomial terms (X_1_^2^ and X_2_^2^) are included to find nonlinearity. The fitted equations to various responses are given below. Y_1_=3.22−0.5X_1_−0.83X_1_^2^+0.166X_2_^2^ (F=15.9, DF=8)-(2), Y_2_=41.44−7.33X_1_+6.0X_2_+7.5X_1_X_2_+11.33X_1_^2^−1.66X^2^ (F=3.8, DF=8)-(3), Y_3_=32.0−0.33X_1_+4.0X_2_−0.5X_1_X_2_−2.0X_1_^2^+1.0X_2_^2^ (F=1.036, DF=8)-(4). The values of correlation coefficients are 0.9816, 0.9298 and 0.8012 respectively. Absence of coefficient X_1_ X_2_ in Eqn. 2 indicates non-significance of both independent variables to hardness. High positive value of X_1_X_2_ coefficient in Eqn. 2 indicates beneficial or synergistic interaction to disintegration time. The low value of X_1_X_2_ coefficient in Eqn. 4 also suggests that the interaction between X_1_ and X_2_ is not so significant. This might be due to high solubility of pectin and polyethylene glycol in water. Optimization by factorial design showed that formulation B_4_ has maximum hardness, minimum disintegration time and minimum t_80_ value as shown in Table 6. Evaluation of preblend of drug and excipients as shown in Table 7 showed that formulation containing either xylitol or dextrose alone (Formulation B_1_, B_2_, B_3_ and B_7_, B_8_ and B_9_) have better flowability and percentage compressibility than the mixtures of xylitol and dextrose (B_4_, B_5_, B_6_). This might be due to decreased homogeneity of the blend due to absorption of moisture by individual components[38]. Characteristics of the tablet prepared have been listed in the same table. The friabilities of all formulations are within the USP limit (0.5-1.0%). Thermoplastic nature of the granules is such that they are less prone to fragmentation[39]. Porosity values showed close similarity in all the formulations. This is because no technique is used to increase pores in the tablet matrix and disintegration is purely based on the low melting point component and sugar based excipients. This was confirmed by the values of *in vitro* and *in vivo* disintegration time and water absorption ratio. It was found that there is a positive correlation between wetting and disintegration time. Percentage ambroxol absorbed from the mucous membrane varied from 15-18%. Unionized nature of the basic drug at the salivary pH could be the reason behind this. Comparison of dissolution data of conventional tablet and fast melt tablets of ambroxol hydrochloride in 0.1N HCl medium showed that there is not much change in the dissolution rate although fast melt tablet showed decrease in dissolution initially as shown in fig. 3. This indicated that compression pressure does not affect the rate of dissolution as confirmed by the similar release pattern from non-compressed pectin microspheres. Finally sensory study on disintegration time and mouth feel attributes ranked the present formulation based on grittiness, chalkiness and overall preference as the best. It can be concluded from the present work that encapsulating the drug in water soluble polymer like pectin could significantly reduce the bitter taste without compromising dissolution rate. Fast melt tablet provides an excellent mouth feel and good physical stability since it melts at about 37°. This dosage form is convenient, economically feasible and needs only a modification of the conventional tableting method.

**TABLE 7:** CHARACTERISTICS OF PREPARED TABLETS

Properties	B_1_	B_2_	B_3_	B_4_	B_5_	B_6_	B_7_	B_8_	B_9_
Angle of repose (θ)	25.86	26.94	26.54	29.76	28.74	29.94	27.54	25.67	26.23
	0.623	0.321	0.124	0.398	0.231	0.114	0.342	0.162	0.215
Compressibility (%)	24.67	26.76	27.32	29.32	28.88	29.43	26.54	25.52	27.65
	0.456	0.327	0.226	0.438	0.198	0.217	0.179	0.146	0.210
Friability (%)	0.765	0.789	0.801	0.567	0.623	0.774	0.745	0.732	0.724
	0.119	0.123	0.131	0.113	0.114	0.124	0.126	0.126	0.127
Porosity (%)	11.32	12.13	12.18	11.98	11.76	11.95	12.01	12.31	12.43
	0.011	0.012	0.014	0.011	0.014	0.015	0.015	0.016	0.016
Wetting time (sec)	77.0	58.0	65.0	43.0	52.0	50.0	41.0	58.0	57.0
	4.56	3.89	2.76	3.98	4.56	4.12	3.32	3.14	3.56
Water absorption ratio (sec)	77.56	86.96	79.93	92.12	89.97	90.12	93.48	87.71	88.98
	4.67	5.32	6.76	4.32	5.68	7.76	3.34	4.67	4.36
*In vivo* disintegration time (sec)	67.0	55.0	58.0	32.0	48.0	50.0	37.0	58.0	58.0
	4.43	3.98	4.12	3.27	5.12	2.67	3.33	4.12	4.76
% Ambroxol absorbed from buccal cavity.	18.12	17.58	17.77	18.43	16.76	17.34	17.01	15.55	15.02
	1.68	1.23	1.59	1.98	1.69	1.46	1.76	2.01	2.79

Physical characteristics such as friability, porosity, wetting time, water absorption ratio and *in vivo* disintegration time and percentage absorption of drug of prepared tablets. Each value is the mean with CV
